# Development of Real-Time Transendothelial Electrical Resistance Monitoring for an In Vitro Blood-Brain Barrier System

**DOI:** 10.3390/mi12010037

**Published:** 2020-12-30

**Authors:** Kai-Hong Tu, Ling-Shan Yu, Zong-Han Sie, Han-Yi Hsu, Khuloud T. Al-Jamal, Julie Tzu-Wen Wang, Ya-Yu Chiang

**Affiliations:** 1 Department of Mechanical Engineering, National Chung Hsing University, Taichung 402701, Taiwan; tcjason2016@gmail.com (K.-H.T.); iopo13465252@gmail.com (Z.-H.S.); fylt34828ms@gmail.com (H.-Y.H.); 2 Rapid Screening Research Center for Toxicology and Biomedicine, National Sun Yat-sen University, Kaohsiung 813016, Taiwan; lingshanyu@mail.nsysu.edu.tw; 3 School of Cancer and Pharmaceutical Sciences, Faculty of Life Sciences & Medicine, King’s College London, London SE5 9NU, UK; khuloud.al-jamal@kcl.ac.uk (K.T.A.-J.); julie.tzu-wen.wang@kcl.ac.uk (J.T.-W.W.); 4 i-Center for Advanced Science and Technology, National Chung Hsing University, Taichung 402701, Taiwan

**Keywords:** transendothelial electrical resistance, TEER, in-situ monitoring, 3D cell culture

## Abstract

Three-dimensional (3D) cell cultures and organs-on-a-chip have been developed to construct microenvironments that resemble the environment within the human body and to provide a platform that enables clear observation and accurate assessments of cell behavior. However, direct observation of transendothelial electrical resistance (TEER) has been challenging. To improve the efficiency in monitoring the cell development in organs-on-a-chip, in this study, we designed and integrated commercially available TEER measurement electrodes into an in vitro blood-brain barrier (BBB)-on-chip system to quantify TEER variation. Moreover, a flowing culture medium was added to the monolayered cells to simulate the promotion of continuous shear stress on cerebrovascular cells. Compared with static 3D cell culture, the proposed BBB-on-chip integrated with electrodes could measure TEER in a real-time manner over a long period. It also allowed cell growth angle measurement, providing instant reports of cell growth information online. Overall, the results demonstrated that the developed system can aid in the quantification of the continuous cell-pattern variations for future studies in drug testing.

## 1. Introduction

The process of drug development comprises compound discovery, identification, animal testing, and phase I–III clinical trials [1]. During this process, animal testing and clinical trials consume most of the resources and time [2]. For example, central nervous system disease models are usually set up in vivo because they require a functional blood-brain barrier (BBB) to mimic the anatomical barriers in the brain [3]. However, as there are genetic and physical variations between animals and humans, the observed preclinical results may not be applied directly to humans, and this can hinder the subsequent clinical trials [4]. Besides, animal husbandry requires considerable time and is of high costs [5].

Transwell^®^ plates are often used to form in vitro organ-tissue models. A simplified three-layer design allows multi-cell co-culture and provides a geographically accurate arrangement. Nevertheless, the Transwell^®^ plate cannot simulate the microenvironments of endothelium which constitute capillaries (10–500 μm in diameter) with continuous blood flow. Real organ tissue simulation remains a challenge [6]. In this regard, it is important to develop a more biomimicry-based platform that can facilitate the exploration and understanding of drug penetration mechanisms and thus reduce the cost of animal testing and increase the success rate of clinical trials [7,8,9].

Many recent studies have confirmed that the application of flow shear stress to cells can stimulate cells physically to form growth patterns similar to those noted in vivo [10,11]. In addition, this external flow stimulation enables cells to generate paracellular tight-junction channels and to express tight-junction protein 1, and zonula occludens-1 (ZO-1) [12]. The tight-junction channel formation indicates that the established in vitro model is biomimetic and stable. In addition to the expression of tight-junction proteins, transendothelial electrical resistance (TEER) also represents a critical parameter of cellular barrier tightness. The TEER of paracellular tight-junction channels can be calculated on the basis of a theoretical equivalent circuit and is widely applicable to the quantification of the barrier integrity of the cell layer grown in the Transwell^®^ inserts which permits comparison with the data reported in vivo [13]. The TEER readings are highly dependent on the electrode positions and careful handling of the electrodes is required while introducing them into the Transwell^®^ under test to avoid any disturbance to the cells. The uniformity of the current density generated by the electrodes across the cell layer has a significant effect on the TEER measurements. The symmetrical arrangement of electrodes on both sides of the cell layer/membrane in a microfluidic device generates a more uniform current density across the membrane when compared to conventional TEER chopstick electrodes.

In this study, we have demonstrated a three-layered microfluidic chip with the custom-made electrodes placed symmetrically in the chip and connected to a TEER measurement device for online real-time monitoring. Fluids were also infused continuously in the microfluidic chip to simulate the actual shear stress occurring in the in vivo capillaries. The real-time TEER monitoring results of this dynamic culture method were investigated.

## 2. Materials and Methods

### 2.1. In Vitro Blood-Brain Barrier (BBB) Model and Principles of Substance Penetration

**Figure 1:**
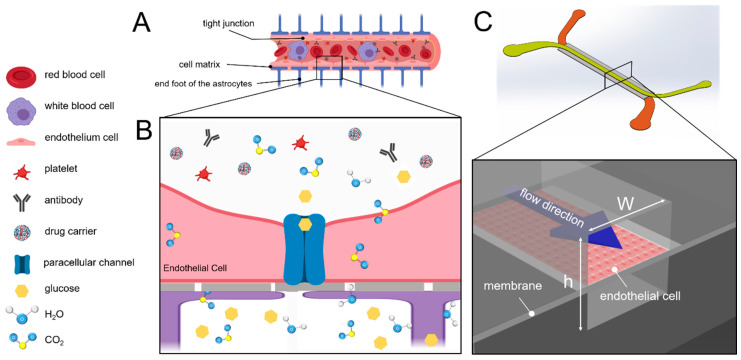
Schematic of the blood-brain barrier (BBB). (**A**) the central endovascular cavity is surrounded by layers outward; in order, they are endothelial cell, extracellular matrix, and astrocyte synapse layers. This structure enables the BBB to filter substances thoroughly. (**B**) schematic of the mechanism by which substances penetrate the BBB. (**C**) schematic of the channel. The purple arrow represents the flow direction. The upper surface of the channel is composed of endothelial cells, whereas the lower surface comprises cultured astrocytes (not included in this experiment). The closer the fluid is to the wall surface, the slower the flow is and the greater the shear stress is.

The BBB is composed of three layers: endothelial cells, an extracellular matrix, and astrocyte glial cells. The endothelial cell layer, which comprises tightly joined cells, has extremely high electrical resistance and is primarily responsible for the filtering of substances. The extracellular matrix layer is composed of connective tissues that limit the activity area of cells and increase cell adhesiveness. The astrocyte layer comprises astrocyte glial cells that form synapses that connect the endothelial cells; the connected astrocytes continuously send signals to the endothelial cell layer so as to form the BBB (Figure 1A [14]. Consequently, only substances that are recognized by the endothelial cell layer, except micro-molecular substances (molecular weight < 1000 Da; e.g., glucose, carbon dioxide, water molecules, and lipid-soluble molecules), can pass through the brain (Figure 1B) [15].

### 2.2. Chip Fabrication and Shear Stress Simulation

During capillary generation, shear stress stimulates cells to alter their growth patterns. The shear stress in the capillary ranges between 4 dyne/cm^2^ and 12 dyne/cm^2^ [12]. In particular, shear stress prompts the secretion of vascular endothelial growth factors in endothelial cells. The growth factors enable the epithelial cells to grow and form capillaries [11]. To simulate the survival of epithelial cells in capillaries, the fluidic shear stress is necessary to the microenvironment (Figure 1C). To control the shear stress that is sustained by cells in the fluidic channel, the flow rate is controlled by using the following equation:

$$\tau_{wall}=\frac{6\mu Q}{wh^{2}}$$

where *w* is the channel width, *h* is the height of the channel, *μ* is the viscosity of the fluid, and *Q* is the input flow rate. Sustained shear stress was assumed to be 0 dyne/cm^2^ and 5 dyne/cm^2^. The flow condition was assumed to be in a steady state, and the thickness of the cells that adhered to the bottom membrane was assumed to be negligible in comparison with the channel height. Thus, when the fluid was in a fully developed flow, τ*_wall_* could be assumed to be the shear stress that was applied to the cells. The width and height of the upper channel were 500 µm and 50 µm, respectively, whereas those of the lower channel were 800 µm and 50 µm, respectively. At the channel inlet and outlet, multiple micropillar structures were designed to prevent dust contamination inside the channel and thus prevent cell inhibition. Polydimethylsiloxane (PDMS, Sil-More industrial Ltd., Taipei, Taiwan) microchannel molds were made through the use of microelectromechanical processes and soft lithography.

**Figure 2:**
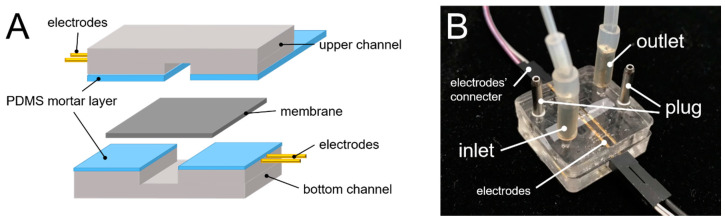
The fabrication of the microchip. (**A**) schematic of electrodes (protruding structures) with the membrane placed between the channels and (**B**) photograph of the integrated system.

Polyethylene terephthalate (PET) membranes of Transwell^®^ inserts were cut into 3 × 10 mm^2^ rectangles, to be placed in the middle of the upper and lower channels. PDMS was used to glue the upper and lower microfluidic channel molds (Figure 2A). PDMS and a curing agent (10:1 w/w) were mixed with pure toluene in a 1:1 ratio [16] and the resultant mixture was used as an adhesive between the two molds. Uniform PDMS-toluene adhesive application was achieved by a thin-film PDMS microcontact printing method as described previously [17]. The channels and the PET membranes were then joined to each other and baked in an oven for 50 °C for 2 h (Figure 2B).

To ensure that the flow velocity and shear stress in the system conformed to the theoretical values, the flow field was analyzed using the micro-particle image velocimetry (µ-PIV) method and analyzed using MATLAB-embedded software, PIV-Lab. The movement of fluorescent particles (3.2 μm, Fluoro-Max^TM^ red fluorescent, Thermo, Hemel Hempstead, UK) was recorded under a 1000 fps high-speed camera. The distance that a particle had moved in two consecutive photographs was determined as the particle movement in unit time. COMSOL 5.5 multiphysics software was applied to provide a theoretical estimate of the flow field velocity.

### 2.3. TEER Measurement

**Figure 3:**
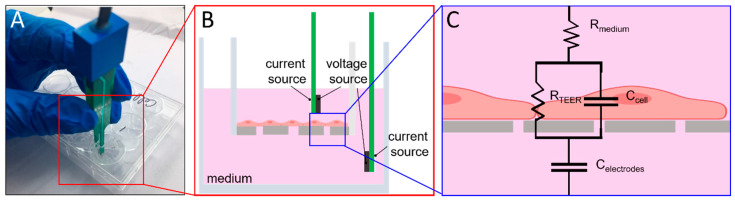
TEER measurement circuit hypothesis and Transwell^®^ plate measurement. (**A**) photograph of the actual Transwell^®^ plate and how TEER measurement is done. (**B**) electrode arrangement of EVOM^2^, (**C**) TEER measurement circuit; the resistances of cell growth medium and membrane are denoted by R_medium_ and R_membrane_, respectively. R_membrane_ is extremely high; therefore, the parallel connection of the R_membrane_ and C_cell_ can be simplified as C_cell_ [18]. C_electrodes_ is the capacitance of the electrode for resistance measurement, and R_TEER_ is the resistance of the paracellular tight-junction channel, which is the primary measurement target in this experiment.

The electrode measurement principle is illustrated in Figure 3. A commercial volt/ohm meter for epithelial cells (EVOM^2^, WPI, Sarasota, FL, USA) relayed alternating current signals through chopstick electrodes (voltage at 12.5 Hz). The two voltage and current sources could become emitters and receivers interchangeably during the current alternation. Through variation in voltage and current signals, resistance was calculated. When the resistance of the paracellular tight-junction channel (R_TEER_) increased, the measured TEER increased. In combination with the EVOM^2^, the electronic component pins (gold-sputtered nickel electrode) were used as electrodes. Each home-made electrode was 0.6 mm in width and the paired electrodes were inserted and aligned into the upper and lower channels. The liquid contact area of each home-made electrode was approximately 0.315 mm^2^. The electrodes on the same side of the chip were placed 2 mm apart. The distance between the upper and lower electrodes was 100 µm. The integrated system was installed as shown in Figure 2B. The same home-made electrodes were also designed for Transwell^®^ configuration with electrodes placed 4 mm apart, which is similar to the conventional STX2 electrode (4.6 mm). All measurements were done in an incubator at 100% relative humidity, 37 °C, and 5% CO_2_ environment.

### 2.4. BBB-On-Chip Culture and Validation

The human cerebral microvascular endothelial cells (hCMEC/D3) were gifts from Prof. Ignacio Romero at Open University, UK. They were used as the in vitro BBB model in this study. All cell cultures were performed using an EGM-2 medium (Lonza, Basel, Switzerland) and only cells from passage 5–10 were used. The device was coated with 100 µg/mL fibronectin before cell loading and then was left to stand in the incubator for 24 h for surface modification. Cells suspended in culture media (10^7^ cells/mL) were first added into the device for 8 h under static culture in order to adhere. The static culture group was maintained in a stationary position while the experimental group was exposed to a flow at 8.3 µL/min in the channel (5 dyne/cm^2^). After the flow stimulation for 24 h, 4% paraformaldehyde (P-6148, Sigma-Aldrich, St. Louis, MO, USA,) was added into the channel to facilitate fixation and subsequent staining and imaging. 4′,6-diamidino-2-phenylindole (DAPI) (nucleus, 1:1000, Thermo Scientific, Bremen, Germany), along with mouse anti-human ZO-1 antibodies (ZO-1 surface antigen of paracellular tight-junction channel, 1:200, BD Biosciences, San Diego, CA, USA) and fluorescein isothiocyanate (FITC)-tagged goat anti-mouse FITC antibodies (primary antibody mark, 1:250, BD Pharmingen^TM^, San Diego, CA, USA), were used for staining. The PTE membrane was then moved from the Transwell^®^ plate and mounted between two thin cover-slips for further inspection. The morphology of the stained cells was imaged using a confocal microscope (FV3000, Olympus, Tokyo, Japan). The cell orientation was then marked and analyzed per interval of 10° with flow field direction using ImageJ. The TEER was monitored at an interval of 6 h for a period of 120 h to observe variations after the cells had sustained shear stress and external interruptions such as a scratch on the cell monolayer.

## 3. Results and Discussion

### 3.1. µ-PIV Flow-Field Distribution Result

**Figure 4:**
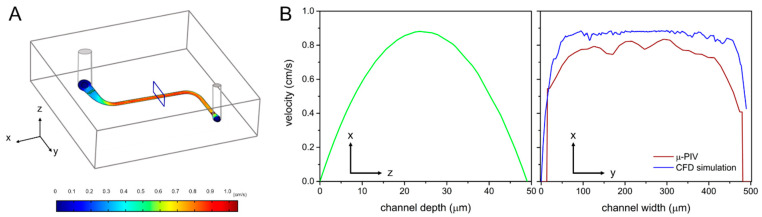
Simulation results of the flow field. (**A**) a flow-field analysis was performed on the straight line area in the center of the channel, and its results were used to predict the shear stress on cells. Estimated distributions of (**B**) velocity in the channel, based on COMSOL simulation and µ-PIV velocity analysis.

COMSOL simulation and µ-PIV velocity analysis results showed maximum velocities of 0.0089 m/s and 0.0083 m/s, respectively (Figure 4). It was decided that the cells might have sustained similar amounts of shear stress to those that were estimated theoretically. The results also confirmed that microchannels simulated a micro-environment that was close to the in vivo.

### 3.2. EVOM^2^ STX2 Electrode vs. Home-Made Electrode

**Figure 5:**
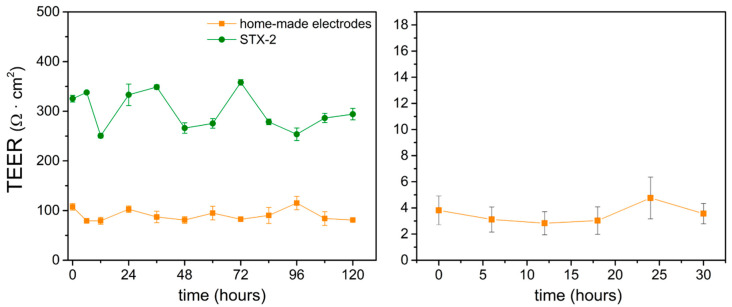
TEER measurement comparison of standard EVOM^2^ STX2 electrodes and home-made electrodes in the Transwell^®^ plate (**left**) and microfluidic chip (**right**). *n* = 3.

The home-made electrode TEER measurements were done in the conventional Transwell^®^ plates and in micro-channels and compared with the standard EVOM^2^ STX2 electrode. Long-term observation was conducted to determine the stability of the electrodes in the incubator. Measurements were taken at 6 h intervals for 120 h and 30 h in Transwell^®^ plates and chips, respectively. The measurements revealed that the home-made electrode-measured values were lower than standard EVOM^2^ STX2. The disparity TEER values may result from the difference of physical dimensions between the STX2 and home-made electrodes in both Transwell^®^ plate and chip configurations, including electrodes’ material, liquid contact area, and the depth of dipping in the targeting substance (R = ρL/A, where ρ is resistive, L is the distance between the electrodes, and A is the liquid contact area). These factors can lead to different TEER values between the home-made electrode and STX2 electrode. However, the electrodes showed a similar trend to the standard EVOM2 STX2 electrode and presented stable values during the experimental period. The TEER values measured by the home-made electrode varied within the range of 17–25 Ω on 4.67 cm^2^ (80–120 Ω·cm^2^) and showed a similar trend to the standard EVOM^2^ STX2 electrode (Figure 5, left). Compared with our home-made electrode, measurements done by the STX2 electrode resulted in significant value variation, possibly due to the gesture error of using STX2: the electrode positions could not be defined precisely and this may have caused the fluctuations. In the microfluidic device, the results measured by the home-made electrodes (effective area of 0.05 cm^2^) also indicated stable TEER monitoring (Figure 5, right).

**Figure 6:**
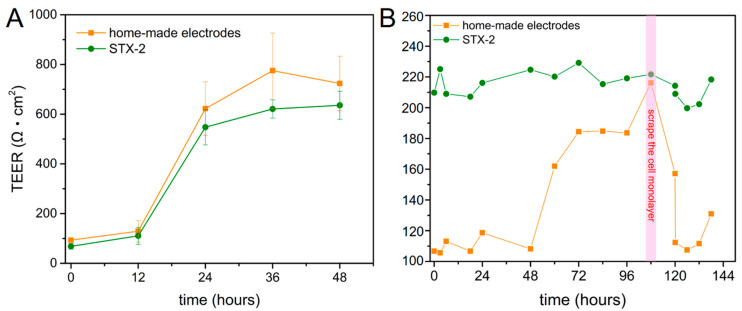
TEER in (**A**) 6 and (**B**) 12-well Transwell^®^ plates. Red area represents the time-point at which cell monolayers were physically damaged.

The TEER value reflects the physical structures and properties of endothelial cultures. We monitored the TEER variation of cells that were seeded on 6 and 12-well Transwell^®^ plates (effective areas were 4.67 and 1.12 cm^2^, respectively). The result showed that TEER value increase with cell proliferation on the membrane in 48 h (Figure 6A). Another experiment demonstrated that when cells reached maximum confluence, a scraper was used to disrupt the cell monolayer integrity; this aided in the measurement of the sharply decreasing TEER value. Subsequently, the TEER continued to rise back to 126 Ω·cm^2^ and the home-made electrode immediately recorded the recovery of the monolayer’s integrity (Figure 6B). It is important to note that the TEER value is reported in units of different effective areas of 6 and 12-well Transwell^®^ plates.

### 3.3. Shear Stress-Assisted Cell Growth

**Figure 7:**
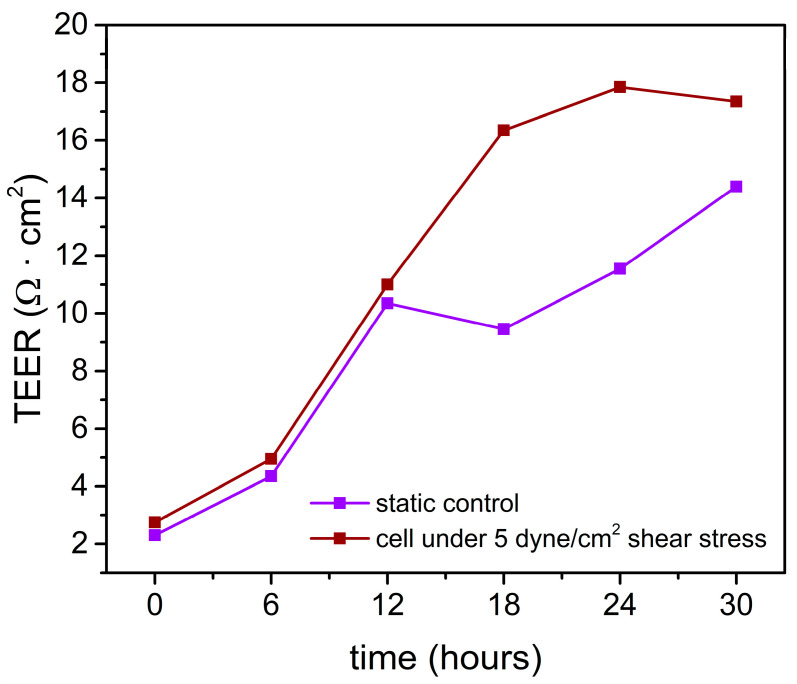
TEER value measurement in the chip using home-made electrodes. Shear stress was applied to the cells at the 12th hour.

The monolayer cell resistance on the chip was monitored and the result showed that TEER increased gradually during the cultivation (Figure 7). The data revealed that before and during exposure to shear stress, the experimental and control groups exhibited minimal differences. However, after shear stress exposure, cell resistance continued to increase in the experimental group to a maximum value of 17.35 Ω·cm^2^. Although cell resistance increased gradually to 14.4 Ω·cm^2^ in the control group (no shear stress added), the monolayer resistance remained lower than that of cells placed under shear stress. The TEER significance of this response to shear stress is quite evident if one looks at the cell morphological change. A barrier selectivity study could be performed to confirm the tightness of the corresponding cell monolayer [19].

**Figure 8:**
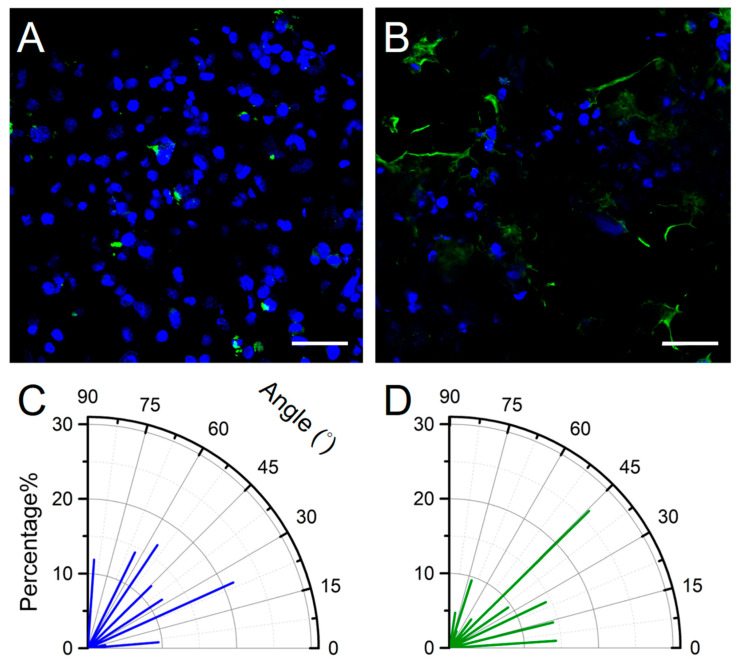
Cellular immunofluorescence staining and angle calculation. (**A**) no ZO-1 expression is visible in cells not exposed to shear stress, and (**B**) ZO-1 expression (green) is visible in cells under shear stress as documented using a confocal laser scanning microscope (DAPI: blue). Cell growth angles for cells under (**C**) no shear stress and (**D**) 5 dyne/cm^2^ shear stress for one day (*n* = 42).

To further quantify the effect of flow on cell growth, we used computer-aided design (CAD) software to analyze the angle between the cell growth angle (θ°) and the flow orientation as well as the presence of cell pattern variation; thus, we determined the effect of shear stress on cell growth. The angle measurement method is reported in previous work [20]. Compared with the aforementioned static culture, the cells in the chip sustained shear stress of 5 dyne/cm^2^ for 24 h. The staining results revealed that the cells expressed ZO-1 characteristics after one-day exposure to shear stress, whereas no ZO-1 expression was noted in cells that had not been exposed to shear stress. The findings confirmed shear stress promoted paracellular tight-junction channel generation in the current design of BBB-on-chip (Figure 8). It is important to note that the present imaging strategies are to facilitate the measurements of cell growth angles. Due to the wrinkled membrane, imaging of the cell monolayer at multiple focal planes was also performed to provide better observation of the cell growth and development (Supplementary Materials Video S1).

The cell orientation angle can be randomized from 0° (parallel to the flow direction) to 90° (perpendicular to the flow direction). Based on the central limit theorem, random cell growth angles (discrete events) should have an approximately normal distribution. We considered that cells cultured on a plain plate without any external cue have a mean random growth angle of 45°, which is used as the reference value in this study. The cell growth angle is a crucial factor that can indicate the influence of shear stress on cells. To further quantify the effect of shear stress on the cell monolayer, we used computer-aided design (CAD) software to analyze the angle between the cell growth angle (θ°) and the flow direction as well as the presence of cell pattern variation (Figure 8C,D). The average growth angle of cells cultured under no shear stress was 43.6°, whereas, under shear stress, it was 36.0°. A mean random growth angle of 45° was used as the reference value: cells under no shear stress exhibiting a <45° growth angle should be approximately 50%. The slightly aligned cells were present under shear stress. The amount of cell orientation less than 45° increased to 64.2% for cells that grew under shear stress for one day. This observation has two implications. First, the major cell axis shows the orientation with the direction of flow after the application of 5 dyne/m^2^ shear stress for 24 h. Second, low expression of tight junction ZO-1 proteins was observed even with a nonconfluent cell monolayer. However, the cell’s cytoskeletal system was not discussed in this study. Analyses of the occurrence and distribution of stress fiber may further support the hypothesis of cytoskeletal system remodeling by fluid-imposed shear stress. Nevertheless, this study shows a primary result of using low-cost home-made electrodes for possible on-line TEER monitoring in the application of organ-on-chip.

These results suggest that the TEER readings are highly dependent on the electrode positions and careful handling of the electrodes is required. The factors affecting TEER measurement have been shown to be temperature [21,22,23], cell line, passage number, culture period [24,25,26,27], medium composition [28,29], and mechanotransductive effect [30,31,32] dependent. The home-made electrode presented in this work has a similar TEER measuring trance to the commercial electrode in the Transwell^®^ plate experiment and good stability that is stable due to the continuous measurement in the incubator directly. Moreover, the TEER values measured in vivo have been reported to be as high as 5900 Ω·cm^2^ [33]. Even the human brain endothelial cell lines (hCMEC/D3) used in co-culture with astrocytes have yielded relatively low TEER value and less than 500 Ω·cm^2^ TEER value is measured in currently available in vitro models [34,35,36,37,38,39,40]. In this study, we demonstrated that a microfluidic platform can allow the exposure of cells to dynamic conditions that provide 5 dyne/cm^2^ shear stress simulation. The integration of home-made electrodes within these systems would allow continuous monitoring of cells without disrupting the cell structure. Our home-made electrode showed stable TEER monitoring in the microfluidic channel and has a fast response to the change of cell monolayer integrity. The disparity in TEER values between other studies may result from the difference in physical dimensions. Since TEER values are accepted as strong indicators of the integrity of cellular barriers, we believe that universal TEER electrode conversion formulas in regard to the material, the geometry of the electrode pad, the distance between the electrode, and effective area need to be set for comparison [41].

## 4. Conclusions

In this study, we used a microchannel structure that involved a continuous flow of fluids through it to simulate the shear stress that was sustained by capillary surfaces. Cells under such physical stimulation were able to form paracellular tight junctions that showed characteristics similar to those found in vivo. Moreover, home-made electrodes were placed delicately in the microfluidic chips and in the Transwell^®^ plate to measure TEER. Real-time cell resistance was measured to quantify cell growth patterns in vitro. Flow field analysis of the experimental environment, involving the use of COMSOL and µ-PIV, was performed to simulate and determine actual flow velocity and shear stress.

The results revealed a <10% error between the simulated and measured values of the maximum flow velocities and shear stress; moreover, the actual flow velocity and shear stress distribution were similar to those that were calculated by use of theory. Cell growth angles were also affected by shear stress; the inclination of the cells was in the direction of the fluids. Thus, the stimulation caused by fluid shear stress affected cell growth. Additionally, gold-coated electrodes exhibited favorable stability. The measured values indicated that fluid shear stress applied to cells resulted in tight patterning.

The in vitro BBB-on-chip that is proposed in this study could be applied to real-time resistance measurements and thus facilitate the quantification of cell pattern variations, provide drug developers with real-time responses after drug stimulation, and improve the understanding of the mechanisms of action and interaction between drugs and cells. Such a method could expedite and simplify subsequent drug development processes and reduce the costs of drug development considerably.

## Supplementary Materials

The following are available online at https://www.mdpi.com/2072-666X/12/1/37/s1, Video S1: SI Movie 1.

## References

[1] Nicolaou K.C. (2014). Advancing the Drug Discovery and Development Process. Angew. Chem. Int. Ed..

[2] Ferdowsian H.R., Beck N. (2011). Ethical and Scientific Considerations Regarding Animal Testing and Research. PLoS ONE.

[3] Pardridge W.M. (2005). The blood-brain barrier: Bottleneck in brain drug development. NeuroRx.

[4] Liebsch M., Grune B., Seiler A., Butzke D., Oelgeschlager M., Pirow R., Adler S., Riebeling C., Luch A. (2011). Alternatives to animal testing: Current status and future perspectives. Arch. Toxicol..

[5] Akhtar A. (2012). The Costs of Animal Experiments. Animals and Public Health: Why Treating Animals Better Is Critical to Human Welfare.

[6] Huh D., Matthews B.D., Mammoto A., Montoya-Zavala M., Hsin H.Y., Ingber D.E. (2010). Reconstituting Organ-Level Lung Functions on a Chip. Science.

[7] Konar D., Devarasetty M., Yildiz D.V., Atala A., Murphy S.V. (2016). Lung-On-A-Chip Technologies for Disease Modeling and Drug Development. Biomed. Eng. Comput. Biol..

[8] Doke S.K., Dhawale S.C. (2015). Alternatives to animal testing: A review. Saudi Pharm. J..

[9] Sosa-Hernández J.E., Villalba-Rodriguez A.M., Romero-Castillo K.D., Aguilar-Aguila-Isaias M.A., Garcia-Reyes I.E., Hernandez-Antonio A., Ahmed I., Sharma A., Parra-Saldivar R., Iqbal H. (2018). Organs-on-a-Chip Module: A Review from the Development and Applications Perspective. Micromachines.

[10] Damiati S., Kompella U.B., Damiati S.A., Kodzius R. (2018). Microfluidic Devices for Drug Delivery Systems and Drug Screening. Genes.

[11] Ye M., Sanchez H.M., Hultz M., Yang Z., Bogorad M., Wong A.D., Searson P.C. (2014). Brain microvascular endothelial cells resist elongation due to curvature and shear stress. Sci. Rep..

[12] Dela Paz N.G., Walshe T.E., Leach L.L., Saint-Geniez M., D'Amore P.A. (2012). Role of shear-stress-induced VEGF expression in endothelial cell survival. J. Cell Sci..

[13] DeStefano J.G., Xu Z.S., Williams A.J., Yimam N., Searson P.C. (2017). Effect of shear stress on iPSC-derived human brain microvascular endothelial cells (dhBMECs). Fluids Barriers CNS.

[14] Srinivasan B., Kolli A.R., Esch M.B., Abaci H.E., Shuler M.L., Hickman J.J. (2015). TEER Measurement Techniques for In Vitro Barrier Model Systems. J. Lab. Autom..

[15] Daneman R., Prat A. (2015). The blood-brain barrier. Cold Spring Harb. Perspect. Biol..

[16] Greene C., Campbell M. (2016). Tight junction modulation of the blood brain barrier: CNS delivery of small molecules. Tissue Barriers.

[17] Kim M.Y., Kolli A.R., Esch M.B., Abaci H.E., Shuler M.L., Hickman J.J. (2014). Microfabrication of High-Resolution Porous Membranes for Cell Culture. J. Memb. Sci..

[18] Hardelauf H., Frimat J., Stewart J.D., Schormann W., Chiang Y., Lampen P., Franzke J., Hengstler j., Cadenas C., Kunz-Schughart L.A. (2011). Microarrays for the scalable production of metabolically relevant tumour spheroids: A tool for modulating chemosensitivity traits. Lab. Chip.

[19] Cucullo L., Hossain M., Puvenna V., Marchi N., Janigro D. (2011). The role of shear stress in Blood-Brain Barrier endothelial physiology. BMC Neurosci..

[20] Xie Y.-T., Chen J., Chen Y., Jiang B., Sie Z., Hsu H., Chen T., Chiang Y., Hsueh H. (2020). Sol–gel-derived hierarchically wrinkled mesoporous ceramics for enhancement of cell alignment. Chem. Eng. J..

[21] Torres R., Pizarro L., Csendes A., García C., Lagos N. (2007). GTX 2/3 epimers permeate the intestine through a paracellular pathway. J. Toxicol. Sci..

[22] Blume L.F., Denker M., Gieseler F., Kunze T. (2010). Temperature corrected transepithelial electrical resistance (TEER) measurement to quantify rapid changes in paracellular permeability. Pharmazie.

[23] Konsoula R., Barile F.A. (2005). Correlation of in vitro cytotoxicity with paracellular permeability in Caco-2 cells. Toxicol. In Vitro.

[24] Lu S., Gough A.W., Bobrowski W.F., Stewart B.H. (1996). Transport properties are not altered across Caco-2 cells with heightened TEER despite underlying physiological and ultrastructural changes. J. Pharm. Sci..

[25] Briske-Anderson M.J., Finley J.W., Newman S.M. (1997). The Influence of Culture Time and Passage Number on the Morphological and Physiological Development of Caco-2 Cells. Proc. Soc. Exp. Biol. Med..

[26] Ponce de León-Rodríguez M.d.C., Guyot J.-P., Laurent-Babot C. (2019). Intestinal in vitro cell culture models and their potential to study the effect of food components on intestinal inflammation. Crit. Rev. Food Sci. Nutr..

[27] Sambuy Y., De Angelis I., Ranaldi G., Scarino M.L., Stammati A., Zucco F. (2005). The Caco-2 cell line as a model of the intestinal barrier: Influence of cell and culture-related factors on Caco-2 cell functional characteristics. Cell Biol. Toxicol..

[28] Ferruzza S., Rossi C., Sambuy Y., Scarino M.L. (2013). Serum-reduced and serum-free media for differentiation of Caco-2 cells. Altex.

[29] Ferruzza S., Rossi C., Scarino M.L., Sambuy Y. (2012). A protocol for differentiation of human intestinal Caco-2 cells in asymmetric serum-containing medium. Toxicol. In Vitro.

[30] Seebach J., Dieterich P., Luo F., Schillers H., Vestweber D., Oberleithner H., Galla H.J., Schnittler H.J. (2000). Endothelial barrier function under laminar fluid shear stress. Lab. Investig..

[31] Béguin E.P., Janssen E.F.J., Hoogenboezem M., Meijer A.B., Hoogendijk A.J., van den Biggelaar M. (2020). Flow-induced Reorganization of Laminin-integrin Networks Within the Endothelial Basement Membrane Uncovered by Proteomics. Mol. Cell Proteom..

[32] Fang Y., Wu D., Birukov K.G. (2019). Mechanosensing and Mechanoregulation of Endothelial Cell Functions. Compr. Physiol..

[33] Butt A.M., Jones H.C., Abbott N.J. (1990). Electrical resistance across the blood-brain barrier in anaesthetized rats: A developmental study. J. Physiol..

[34] Griep L.M., Wolbers F., de Wagenaar B., ter Braak P.M., Weksler B.B., Romero I.A., Couraud P.O., Vermes I., van der Meer A.D., van den Berg A. (2013). BBB on chip: Microfluidic platform to mechanically and biochemically modulate blood-brain barrier function. Biomed. Microd..

[35] Daniels B.P., Cruz-Orengo L., Pasieka T.J., Couraud P., Romero I.A., Weksler B., Cooper J.A., Doering T.L., Klein R.S. (2013). Immortalized human cerebral microvascular endothelial cells maintain the properties of primary cells in an in vitro model of immune migration across the blood brain barrier. J. Neurosci. Methods.

[36] Biemans E.A.L.M., Jakel L., de Waal R.M.W., Kuiperij H.B., Verbeek M.M. (2017). Limitations of the hCMEC/D3 cell line as a model for Aβ clearance by the human blood-brain barrier. J. Neurosci. Res..

[37] Weksler B., Romero I.A., Couraud P.-O. (2013). The hCMEC/D3 cell line as a model of the human blood brain barrier. Fluids Barriers CNS.

[38] Gericke B., Romermann K., Noack A., Noack S., Kronenberg J., Blasig I.E., Loscher W. (2020). A face-to-face comparison of claudin-5 transduced human brain endothelial (hCMEC/D3) cells with porcine brain endothelial cells as blood–brain barrier models for drug transport studies. Fluids Barriers CNS.

[39] Booth R., Kim H. (2012). Characterization of a microfluidic in vitro model of the blood-brain barrier (μBBB). Lab. Chip.

[40] Henry O.Y.F., Villenave R., Cronce M.J., Leineweber W.D., Benz M.A., Ingber D.E. (2017). Organs-on-chips with integrated electrodes for trans-epithelial electrical resistance (TEER) measurements of human epithelial barrier function. Lab. Chip.

[41] Yeste J., Illa X., Gutierrez C., Sole M., Guimera A., Villa R. (2016). Geometric correction factor for transepithelial electrical resistance measurements in transwell and microfluidic cell cultures. J. Phys. D Appl. Phys..

